# Exploiting syntactic and semantics information for chemical–disease relation extraction

**DOI:** 10.1093/database/baw048

**Published:** 2016-04-14

**Authors:** Huiwei Zhou, Huijie Deng, Long Chen, Yunlong Yang, Chen Jia, Degen Huang

**Affiliations:** School of Computer Science and Technology, Dalian University of Technology, Dalian 116024, People's Republic of China

## Abstract

Identifying chemical–disease relations (CDR) from biomedical literature could improve chemical safety and toxicity studies. This article proposes a novel syntactic and semantic information exploitation method for CDR extraction. The proposed method consists of a feature-based model, a tree kernel-based model and a neural network model. The feature-based model exploits lexical features, the tree kernel-based model captures syntactic structure features, and the neural network model generates semantic representations. The motivation of our method is to fully utilize the nice properties of the three models to explore diverse information for CDR extraction. Experiments on the BioCreative V CDR dataset show that the three models are all effective for CDR extraction, and their combination could further improve extraction performance.

**Database URL**: http://www.biocreative.org/resources/corpora/biocreative-v-cdr-corpus/.

## Introduction

Understanding the relations between chemicals and diseases is relevant to many areas of biomedical research and health care, e.g. drug discovery and safety surveillance (1). Biomedical researchers have studied a great amount of associations between chemicals and diseases, and published their studies in the biomedical literature. However, manually extracting these relations is expensive and time-consuming, and it is impossible to keep up-to-date. Automated natural language processing (NLP) methods could extract the chemical–disease relation (CDR) to keep pace with the fast growth of biomedical literature.

The BioCreative V (2) proposes a challenge task of automatic CDR extraction from the biomedical literature by text mining technique. There are two specific subtasks: (i) disease named entity recognition and normalization (DNER) and (ii) chemical-induced diseases relation extraction (CID). This paper focuses on the CID subtask. For the task, a total of 1500 PubMed articles (3): 500 each for the training, development and test set are prepared.

Previous research on relation extraction (RE) can be divided into two categories: rule-based methods and machine learning-based methods. Rule-based methods extract CDR by adopting prototypical relation patterns. Lowe *et al.* (4) develop a simple pattern-based system to find chemical-induced disease relations within the same sentence and achieve 52.20% *F*-score on the BioCreative V CDR Task. Rule-based methods could make full use of syntactic information and have achieved good performance in the existing resource, but the extracted rules are hard to develop to a new dataset.

As for machine learning-based RE, feature-based methods and kernel-based methods are widely used. Feature-based methods focus on designing effective features including lexical, syntactic and semantic information. Gu *et al.* (5) utilize rich lexical features for CID task and achieve 55.3% *F*-score on the development set of BioCreative V CDR Task. Bui *et al.* (6) generate flat features from a suitable syntactic structure to improve the performance of drug–drug interaction extraction. Knowledge-based features derived from the database containing prior knowledge about chemicals and diseases are also applied for CDR extraction. Xu *et al.* (7) employ various drug-side-effect resources to generate knowledge-based features, and achieve the highest *F*-score of 57.03% in BioCreative V CDR Task. Pons *et al.* (8) also use knowledge-based features, and get the second best reported result (52.6% *F*-score). Feature-based methods are simple and could achieve good results. However, the traditional lexical and flat syntactic features are ‘one-hot’ representations, which could not adequately capture the deep semantic and syntactic structure information.

Kernel-based methods are more effective than feature-based methods for capturing syntactic structure information, which compute the structure similarity between two trees by tree kernel function (9). The representation of the tree structure is an essential prerequisite for kernel-based methods in state-of-the-art RE systems (10– 12). Zhang *et al.* (10) investigate five tree spans of a phrase tree for general RE task, among which the Path-enclosed Tree (PT) achieves the best performance. The phrase tree represents constituent of neighbors, which is suitable for capturing local syntactic information. Meanwhile, the dependency tree reflects semantic modification relationships of words in a sentence, which compactly represents global syntactic information. To grasp global and local syntactic information connecting chemical and disease entities, Zhou *et al.* (13) integrate phrase and dependency trees to improve the performance for the CDR task.

As for semantic information, deep learning techniques have recently shown to be superior in some NLP tasks. Deep neural networks, such as recurrent neural network (RNN) (14), convolution neural network (CNN) (15, 16) and RNN with long short-term memory (LSTM) units (17), are successfully applied to semantic representations of surface sequences. Liu *et al.* (18) adopt CNN to learn the representation of the shortest dependency path (SDP) between two entities. Nguyen *et al.* (19) demonstrate that semantic representations are effective on the tree kernel-based RE system. They obtain semantic representations of entity pairs by concatenating the word representations of the two entity heads, and use them as features to learn a feature-based model. Xu *et al.* (17) first propose to use LSTM to pick up semantic information along the SDP for RE. LSTM is designed to cope with the gradients vanishing or exploding problem of RNN (20, 21).

Each of the above three machine learning-based methods shows heterogeneous superiority for CDR extraction. This article integrates a feature-based model, a kernel-based model and a neural network model into a unified framework to exploit deep syntactic and semantic information for CDR extraction. Our study shows that surface lexical features with the feature-based model, structured syntactic features with the kernel-based model and semantic representations with the neural network model are all effective for CDR extraction. And their combination could further improve the performance significantly. We especially study how to combine the three models to optimize the performance of the hybrid system.

## Materials and methods

To simplify CDR task, we ignore CDR across sentences and only identify CDR in a sentence. Each chemical–disease pair in a sentence is regarded as a candidate instance. The CDR corpus is preprocessed with GENIA Tagger (http://www.nactem.ac.uk/GENIA/tagger/), Berkeley Parser (http://nlp.cs.berkeley.edu/software.shtml) and Gdep Parser (http://people.ict.usc.edu/~sagae/parser/gdep) to get lexical information, phrase trees and dependency trees, respectively.

**Figure 1.**
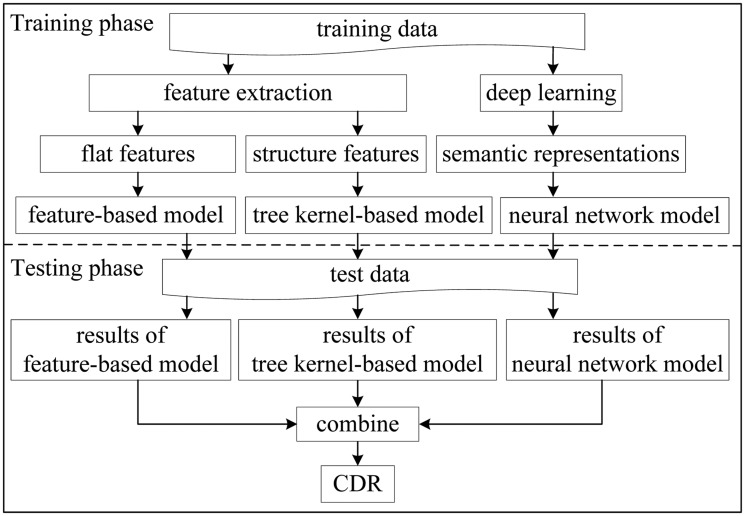
Hybrid system architecture.

The architecture of the hybrid system is shown in Figure 1, which consists of a training phase and a testing phase. In the training phase, we extract flat features and structure features from the training data, and learn semantic representations by deep learning. Thus, the feature-based model, the kernel-based model and the neural network model are obtained. Two categories of neural networks, LSTM and CNN, are used to compute semantic representations of CDR pairs.

In the testing phase, the three models are applied to extract CDR. The predicted results of the three models are combined finally.

### Feature-based model

The feature-based model is learned from flat features with polynomial kernel. We select widely used basic features for CDR extraction as shown below. These features reflect the characteristic of chemical entities, disease entities and their relations between them.

Context: word, stem, POS and chunk of two entities in the window [-3, 3].

Entity: head, POS and chunk.

Position: the positional relationship of two entities. If the chemical entity is before disease, the feature value is set to ‘*before*’. Otherwise the feature value is set to ‘*after*’.

Distance: the number of words between two entities. If there are fewer than three words between two entities, the feature value is set to ‘*LessThree*’. The other feature values include ‘*MoreThreeLessSix*’, ‘*MoreSixLessNine*’, ‘*MoreNineLessTwelve*’ and ‘*MoreTwelve*’.

Verb: if there are verbs before, between and after the two entities.

### Tree kernel-based model

One of the core problems in tree kernel-based RE is how to represent the tree structure. Bunescu and Mooney (22) demonstrate that SDP between two entities could capture the predicate–argument sequences, which provide strong evidence for relation classification. We leverage the shortest dependency path tree (SDPT) to generate structured dependency features (SDF), structured phrase features (SPF) and flattened dependency features (FDF)

#### Shortest dependency path tree

SDPT is the shortest path subtree linking two entities in dependency tree. Taking Sentence 1 as an example, there is a chemical entity denoted by wave line and four disease entities denoted by underline. The chemical entity ‘*fentanyl*’ is associated with the four disease entities.

Sentence 1: *Various reported side effects of fentanyl administration include* ‘*chest wall rigidity*’, ‘*hypotension*’, ‘*respiratory depression*’ *and* ‘*bradycardia*’.

**Figure 2.**
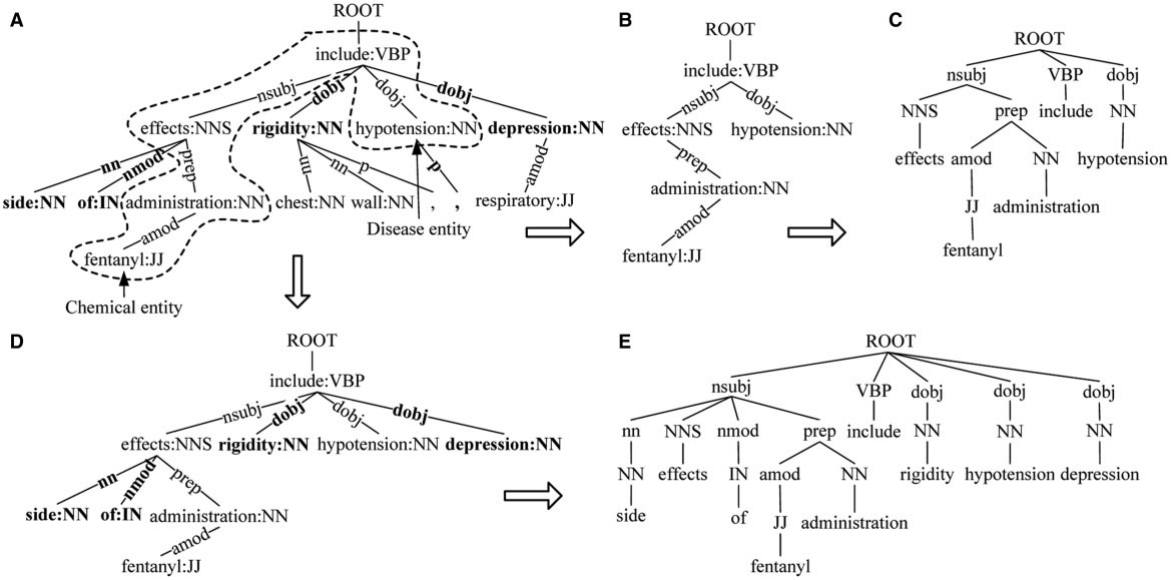
SDPT. (**A**) The fragment of dependency tree for Sentence 1. (**B**) SDPT. (**C**) SDF based on SDPT. (**D**) Extended SDPT. (**E**) Extended SDF based on SDPT.

For the fragment of dependency tree (Sentence 1) shown in Figure 2A, SDPT of the candidate ‘*fentanyl*’ and ‘*hypotension*’ is shown in Figure 2B. SDPT is the most direct syntactic representation connecting the two entities.

#### SDF based on SDPT

For the SDPT shown in Figure 2B, tree kernel cannot capture dependency relation on the arcs (e.g. ‘*dobj*’ relation between node ‘*include*’ and ‘*hypotension*’). To capture dependency relation, we use the dependency relation labels to replace the corresponding word–POS pairs on the nodes of original SDPT as shown in Figure 2C. Then, make the POS tags as the children of the corresponding relation nodes, the fathers of their associated words.

#### Extended SDF based on SDPT

To enrich the context information, SDF is extended with the dependent nodes of all nodes in SDPT to construct extended SDF (shown in Figure 2D and E).

#### SPF based on SDPT

**Figure 3.**
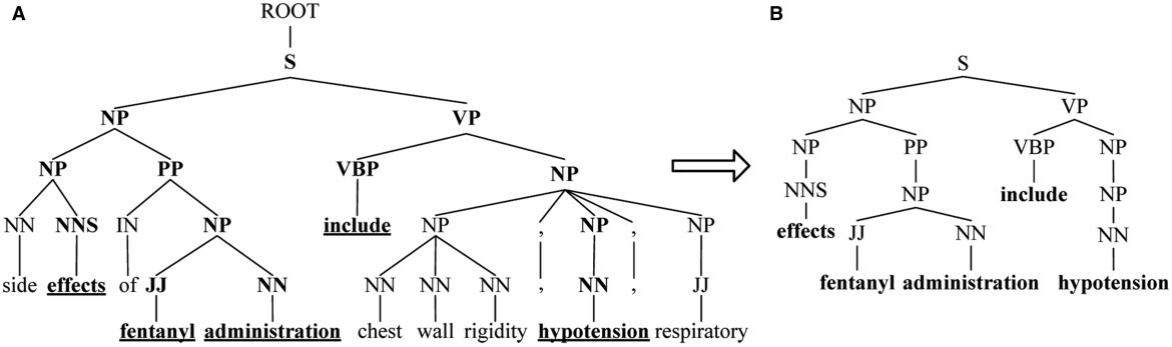
SPF based on SDPT. (**A**) The fragment of phrase tree for Sentence 1. (**B**) SPF based on SDPT.

To capture constituents and exclude redundancy of two entities with long distance, we propose SPF based on SDPT. For the fragment of phrase tree for Sentence 1 shown in Figure 3A, SPF of the candidate ‘*fentanyl*’ and ‘*hypotension*’ is shown in Figure 3B. SPF is a subtree consisting of the words in SDPT (denoted by underline in Figure 3A) and their ancestral constituents (highlighted in bold).

#### FDF based on SDPT

As the root word of SDPT is important for CDR extraction, we use the root features about SDPT as the FDF as follows:

Position: the root word of the SDPT locates before, between or after the two entities.

Context: word, POS and chunk features in the window [-1, 1].

### Neural network model

**Figure 4.**
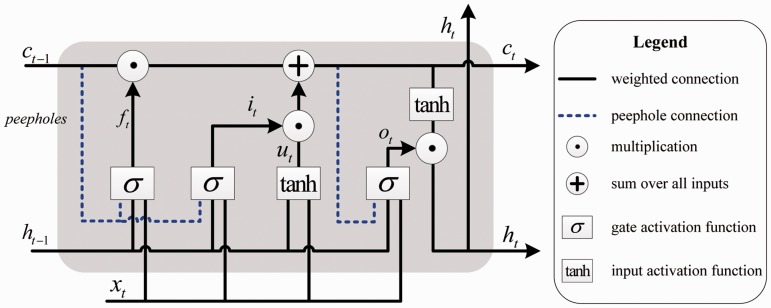
Detailed architecture of the peephole LSTM.

Specifically, we use LSTM to generate semantic representations of CDR pairs. LSTM introduces a gating mechanism, which comprises four components: an input gate *i_t_*, a forget gate *f_t_*, an output gate *o_t_* and a memory cell *c_t_*. For the standard LSTM, each of the three gates receives the information from the inputs at current time step and the outputs at previous time step. Many LSTM variants have been proposed for NLP problems. We adopt a variant, which adds the ‘*peephole connections*’ to the architecture (23) (shown in Figure 4) to let the memory cell *c_t__−_*_1_ directly control the gates as follows:

(1) $$i_{t}=\sigma (W^{\left(i\right)}x_{t}+U^{\left(i\right)}h_{t-1}+V^{\left(i\right)}c_{t-1}+b^{\left(i\right)}),$$

(2) $$f_{t}=\sigma (W^{\left(f\right)}x_{t}+U^{\left(f\right)}h_{t-1}+V^{\left(f\right)}c_{t-1}+b^{\left(f\right)}),$$

(3) ct=ft ⊙ ct−1+it ⊙ tanh⁡(W(c)xt+U(c)ht−1+b(c)),

where *W*, *U* and *V* are the transition matrices for the input *x_t_*, the hidden state vector *h_t__−_*_1_ and the memory cell *c_t__−_*_1_, respectively. *b* is a bias term for the hidden state vector, σ represents the sigmoid function, and ⊙ denotes component-wise multiplication.

The current hidden state value *h_t_* is controlled by the output gate *o_t_*, which is applied to the result of the application of a nonlinearity to the memory cell contents:

(4) $$o_{t}=\sigma (W^{\left(o\right)}x_{t}+U^{\left(o\right)}h_{t-1}+V^{\left(o\right)}c_{t}+b^{\left(o\right)})$$

(5) $$h_{t}=o_{t}{}^{⊙}\tanh(c_{t}).$$

The hidden state *h_t_* at current time step is used for the acquisition of *h_t + _*_1_ at next time step. That is, LSTM processes the word sequence by recursively computing its internal hidden state *h_t_* at each time step. The hidden activations of the last time step could be considered as the semantic representation of the whole sequence and used as input to classification layer.

To explore deep semantic information behind CDR pairs, we adopt the following input methods to learn semantic representations from the surface sequences.

#### WORD

This method inputs the word sequences between chemical and disease entities into LSTM to capture semantic representations of CDR pairs. The dimension of word representations $xw\in R^{d}$ is *d*.

#### WORD-POS

Besides the word sequences, this method additionally inputs POS tags of the word sequences. The representations of each word *w* and its POS *p* are concatenated to form a vector representation $xw,xp\in R^{2}{}^{d}$.

#### HEAD

Compared with WORD, this method replaces all chemical and disease entities with their head words to enhance the generalization capacity. This representation is inherited from Nguyen *et al.* (19) that only concatenate the word representations of the two entity mention heads, whereas our method captures the semantic representation of the whole sequence.

#### SDP-dep

**Figure 5.**
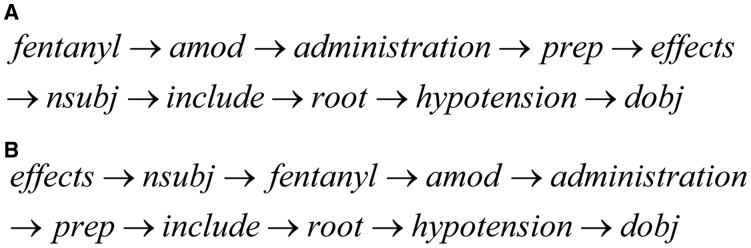
SDP sequences. (**A**) SDP-dep sequence. (**B**) SDP-seq sequence.

This method inputs a sequence of words and dependency relations of SDP as shown in Figure 5A. This is motivated by Liu *et al.* (18), which adopt CNN to learn the semantic representations behind SDP. Note that the sequence follows the left-to-right order in SDP. The dimensions of word representations $xw\in R^{d}$ and relation representation $xr\in R^{d}$ are both *d*.

#### SDP-seq

This method also inputs a sequence of words and dependency relations of SDP. However, the sequence follows the natural order of words in a sentence as shown in Figure 5B. We consider that this order could reflect the actual semantic information in context.

We also try applying CNN to produce semantic representations of CDR pairs. The performance comparison between LSTM and CNN is given in the ‘Results and discussion’ section.

### Hybrid CDR extraction system

We propose a hybrid CDR extraction system integrating the feature-based model *F*(*v_i_*), weighted by α, the tree kernel-based model *K*(*t_i_*), weighted by β, and the neural network model *N*(*s_i_*), weighted by 1−α−β.

The predicted results of the feature-based and tree kernel-based models are the distances between the instances and the separating hyperplanes, whereas those of the neural network model are the probabilities of the test data. We adopt the sigmoid function in our experiments to transform the distance into a probability and extract CDR with a uniform framework:

(6) $$P(R_{i})=\alpha \cdot \sigma (F(v_{i}))+\beta \cdot \sigma (K(t_{i}))+(1-\alpha -\beta )\cdot N(s_{i})$$

where *v_i_*, *t_i_* and *s_i_* are the lexical features, the structure features and semantic representations of the CDR pair *R_i_* in test data, respectively. The parameters α∈[0,1] and β∈[0,1] could be controlled to investigate the impacts of lexical features vs. structure features vs. semantic representations. The sigmoid function is monotonic, and the point P(y=1|f)=0.5 occurs at the separating hyperplanes f=0 (24). Therefore in our experiments, the boundary probability to separate relations from non-relations is simply set to 0.5.

## Results and discussion

Experiments are conducted on the BioCreative V CDR Task corpus. We train the system on the training and the development sets, and evaluate it on the test set. The evaluation of CDR extraction is reported by official evaluation toolkit (http://www.biocreative.org/tasks/biocreative-v/track-3-cdr/), which adopts Precision (*P*), Recall (*R*) and *F*-score (*F*) to measure the performance. SVM-LIGHT-TK toolkit (http://disi.unitn.it/moschitti/Tree-Kernel.htm) is used to construct the feature-based and tree kernel-based models. Neural network model (LSTM model and CNN model) is developed based on Theano system (25). We systematically evaluate the effectiveness of the feature-based model, the tree kernel-based model and the neural network model for CDR extraction. In addition, we investigate their complementarities by combining them with different weighting parameters. Note that all the performances are achieved by using golden standard entities.

### Effects of flat features

The detailed performances of the feature-based model with different flat feature sets are summarized in Table 1. From the results, we can see that:

1. The feature-based model with only context features achieves acceptable results. With other basic features (entity, position, etc.) added one by one, the performance is improved continuously and reaches 53.70% *F*-score. All of the basic features are effective for CDR extraction.
2. When adding the FDF features derived from SDPT, the performance is further improved. However, the improvement is slight. Thus, it can be seen that the flattened syntactic features are helpful for CDR extraction, but they are unable to represent the rich syntactic structure character.

**Table 1.** Performance of the feature-based model with flat features

Flat features	*P* (%)	*R* (%)	*F* (%)
Basic			
Context	59.07	44.00	50.43
+Entity	60.73	45.40	51.96
+Position	60.95	45.68	52.23
+Distance	61.99	46.81	53.34
+Verb	62.15	47.28	53.70
FDF			
+Context	62.39	47.47	53.92
+Position	62.86	47.47	54.09

### Effects of structure features

Table 2 shows the CDR extraction performance of the kernel-based model with structure features. From Table 2, we can see that the sole SDF or sole SPF with tree kernel is comparable to the sole context features. And their combination could improve the performance. These indicate that SDF and SPF are effective and complementary for CDR extraction. Tree kernel-based model can capture useful syntactic structure information inherent in parsing trees.

**Table 2.** Performance of kernel-based model with structure features

Structure features	*P* (%)	*R* (%)	*F* (%)
SDF	57.86	44.18	50.11
SPF	59.08	42.12	49.18
SDF+SPF	59.70	44.18	50.78

We also compare our SDF with the other syntactic structure features, PT (10) and Extended SDF, in Table 3. Both of them perform worse than SDF, which shows that SDF could represent concise as well as precise syntactic structure connecting the two entities.

**Table 3.** Comparison with other structured syntactic representation

Structure features	*P* (%)	*R* (%)	*F* (%)
SDF	57.86	44.18	50.11
PT	63.00	41.37	49.94
Extended SDF	61.17	42.12	49.89

### Effects of semantic representations

In our experiments, the initial word representation is pre-trained by the Word2Vec tool (https://code.google.com/p/word2vec/) (26) instead of randomly sampling. The dimension *d* of Word2Vec is 200, whereas the other parameters are set as default. We first provide the performance of LSTM model to investigate the different input methods as shown in Table 4.

**Table 4.** Performance of LSTM model with the different input methods

Methods	*P* (%)	*R* (%)	*F* (%)
WORD	47.08	56.00	51.16
WORD-POS	52.96	50.28	51.59
HEAD	48.41	55.82	51.85
SDP-dep	50.44	53.85	52.09
SDP-seq	54.08	51.03	52.51
SDP-seq+POS	54.06	51.22	52.60
SDP-seq+HEAD	54.33	51.22	52.73
SDP-seq+POS+HEAD	54.91	51.41	53.10

From Table 4, we can conclude:

The sole WORD with only the word sequences has achieved an acceptable result by learning word representations.

When the POS tags (WORD-POS) are added into the word sequences, the performance improves. The reason may be that POS information could be encoded into word representations and used as additional information.

The generalization of the entities (HEAD) is effective for improving CDR extraction.

The semantic representations based on SDP (SDP-dep, SDP-seq) perform better than those based on the word sequences. This indicates that SDP contains more important information while diminishing less relevant noise. In addition, SDP-seq outperforms SDP-dep, suggesting that the natural order of words is more suitable for LSTM architecture to capture the semantic representation of sequences.

The combination of SDP-seq with either HEAD or POS further improves performance. The best performance is achieved when the HEAD and POS representations are utilized at the same time, reaching an *F*-score of 53.10%. HEAD and POS seem to capture different information.

Then, we experiment another neural network model (CNN model) to produce semantic representations of CDR pairs. The window size and the number of feature maps of convolution layer are set to 3 and 200, respectively. Traditional max-pooling layer is used to capture the most useful information to represent the entity pairs. Experimental results are given in Table 5. It is somewhat disappointing that CNN model does not perform as well as LSTM model, which shows superior power of LSTM in modeling semantic representations of surface sequences.

**Table 5.** Performance of CNN model with the different input methods

Methods	*P* (%)	*R* (%)	*F* (%)
WORD	49.25	46.44	47.80
WORD-POS	46.54	50.47	48.92
HEAD	49.57	48.97	49.27
SDP-dep	42.00	53.66	47.12
SDP-seq	47.64	47.28	47.46
SDP-seq+POS	49.56	47.28	48.39
SDP-seq+HEAD	46.97	48.03	47.50
SDP-seq+POS+HEAD	41.13	55.25	47.16

### Effects of weighting parameters

**Figure 6.**
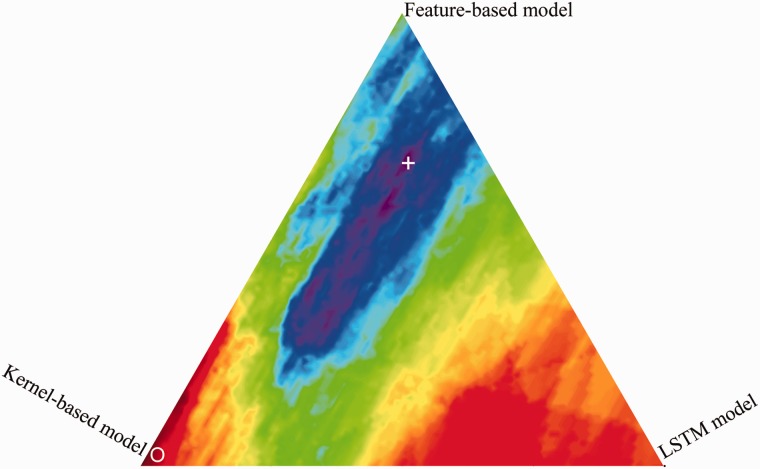
Performance of different weightings of the three models (feature-based model: top, kernel-based model: left, LSTM model: right). ‘**+**’ indicates the maximum; ‘O’ indicates the minimum.

We investigate the impact of the parameters α, β (Hybrid CDR extraction system section) that control the weighting of feature-based model vs. tree kernel-based model vs. neural network model. The weighting parameters of the three models are optimized with a grid search procedure using 5-fold cross-validation experiments, which is conducted on the corpus consisting of training set and development set. The best feature sets of the feature-based and kernel-based models and the best representation method of the LSTM model are used in the hybrid system. From Figure 6, the best performance weighting area (purple) is in the middle, and therefore all the three models are effective for CDR extraction. Apparently, the high weight of feature-based model enables increasing extraction performance. The best performance is obtained with the set of α= 0.68 and β= 0.15. This set of parameters is used in the following experiments for the hybrid extraction system.

Statistical analysis is also performed via 5-fold cross-validation on the corpus consisting of training set and development set. The weighting parameters for the combination of the three models are varied from 0 to 1 with an interval of 0.1. Table 6 reports the average performances of the different weighting parameters over all five cross-validation folds and the *P*-values for comparisons between different combination methods. From the table, we can see that the differences between the combination of the three models (FKL) and that of the two models (FK, FL, KL) are all statistically significant (*P *<* *0.05). The analysis demonstrates that by combining the three models, we can get better syntactic or semantics information for CDR extraction.

**Table 6.** Statistical analysis of different systems. (feature-based, kernel-based and LSTM models are shorted as F, K and L, respectively)

Combination systems	*P* (%)	*R* (%)	*F* (%)	*P*-values
FKL	60.30	49.19	54.18	
FK	64.64	43.94	52.31	0.025
FL	57.36	50.46	53.83	0.032
KL	57.39	50.07	53.48	0.011

### Effects of post-processing

Our hybrid system with the set of α= 0.68 and β= 0.15 is evaluated on the test set. The evaluation result in Table 7 shows that the hybrid system achieves a high precision of 64.89%, but low recall (49.25%). To further pick the most likely CDR, the following two kinds of common post-processing techniques are applied to the results from the hybrid system one by one, and the effects of post-processing are also shown in Table 7.

**Table 7.** Effects of post-processing on the test set

System	*P* (%)	*R* (%)	*F* (%)
Hybrid system	64.89	49.25	56.00
+ Causal relation rules	62.99	51.41	56.61
+ Focused chemical rules	55.56	68.39	61.31

#### Causal relation rules

It is difficult to extract causal relationships between chemicals and diseases by machine learning-based methods. rules to extract causal relations.

- Chemical <related> Disease
- Disease <during> Chemical
- Chemical <caused> Disease
- Chemical <associated> Disease
- Chemical <induced> Disease
- Chemical Disease

#### Focused chemical rules

When no CDR is matched in an abstract, the focused chemical rules is applied to find likely relations.

All chemicals in the title are associated with all diseases in the entire abstract.

When there is no chemical in the title, the most-frequently mentioned chemical in the abstract is associated with all diseases in the entire abstract.

Added post-processing rules to the hybrid system, the recall increases significantly, and the *F*-score is improved from 56.00% to 61.31%. In particular, the focused chemical rules effectively help the hybrid system to pick some missed CDRs from the abstracts where no CDR is found by the hybrid system. As a supplement to the hybrid system, post-processing has a very strong effect.

### Comparison with related work

Table 8 compares our systems with the top three systems in the Biocretive V CDR task. It shows that our system achieves 61.31% *F*-score by using golden standard entities. Compared with the state-of-the-art systems, we recognize the disease and chemical entities with tmChem (27) and Dnorm (28, 29) toolkits, and then use our hybrid system to extract CDR. Our final *F*-score drops to 45.96%, which does not catch up with the performance of the state-of-the-art systems. The highest performance from DNorm requires the UMLS Metathesaurus to provide lexical hints to BANNER and also Ab3P to resolve abbreviations (from the readme.txt of DNorm installation document). However, we do not install the UMLS Metathesaurus successfully. Therefore, quite a few disease names are not recognized or normalized correctly, and the corresponding CDR could not be extracted. By contrast, the top three systems all perform DNER by their own. The results of DNER directly influence the performance of CDR extraction.

**Table 8.** Comparison with related work

System	*P* (%)	*R* (%)	*F* (%)
Ours (golden)	55.56	68.39	61.31
Ours (NER)	42.59	49.91	45.96
Xu *et al.* (7)	55.67	58.44	57.03
Pons *et al.* (8)	51.34	53.85	52.56
Lowe *et al.* (4)	52.62	51.78	52.20

For CDR extraction, Xu *et al.* (7) and Pons *et al.* (8) both use large-scale prior knowledge about chemicals and diseases, and, respectively, achieve the highest *F*-score of 57.03% and the second highest *F*-score of 52.56% in BioCreative V CDR Task. However, our system has not used any external resources. Lowe *et al.* (4) predefine many rules to find CDR simply by a rule-based system. Their system achieves 52.20% *F*-score, but the hand-crafted rules are hard to develop to a new dataset. Compared with these systems, our system is more robust and does not heavily rely on knowledge bases or predefined rules. Our framework makes full use of lexical, syntactic and semantic information, and could be further extended by incorporating other effective information.

### Error analysis

**Figure 7.**
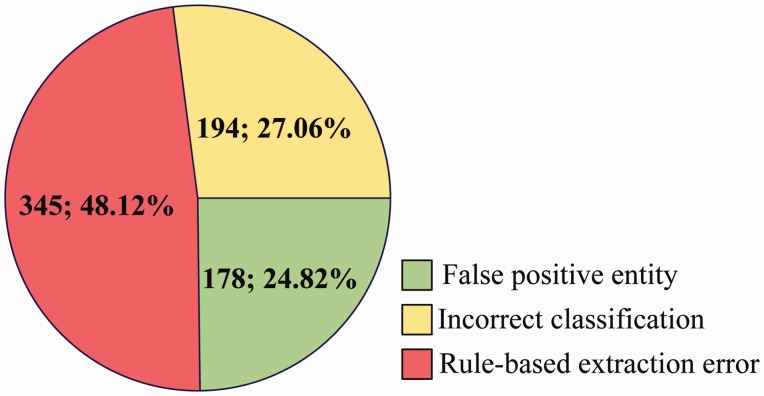
Origins of FP errors.

**Figure 8.**
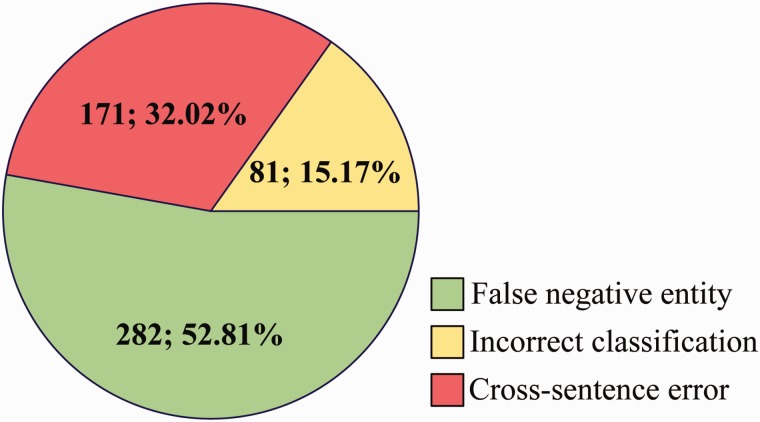
Origins of FN errors.

We perform an error analysis on the output of Ours (NER) (row 2 in Table 8) to detect the origins of false positives (FP) and false negatives (FN) errors, which are categorized in Figures 7 and 8, respectively.

For FP (Figure 7), some main error types are listed as follows:

False positive entity: Among the 717 CDR that are extracted incorrectly, 24.82% is caused by false positive disease or chemical entities, which are not in the gold-standard named entities but recognized by tmChem (26) and Dnorm (27, 28) toolkits.

Incorrect classification: In spite of the rich syntactic structure features and the detailed semantic representations, 27.06% FP come from the incorrect classification made by the three individual models.

Rule-based extraction error: Post-processing rules introduce 345 FP, with a proportion of 48.12%.

For FN (Figure 8), some main error types are listed as follows:

False negative entity: Among the 534 CDR that have not been extracted, 52.81% is caused by false negative entities, which are not recognized by tmChem (26) and Dnorm (27, 28) toolkits.

Incorrect classification: The three single models misclassify 81 positive cases as negatives due to complex syntactic and latent semantic information of entity pairs.

Cross-sentence error: Cross-sentence CDR relation pairs are not extracted in our system. 32.02% FN is caused by span sentence CDRs.

## Conclusions

Lexical features, syntactic structure features and semantic representations are all particularly effective for RE, which can be well captured by feature-based methods, kernel-based methods and deep neural networks, respectively. Different relation classification methods have their own properties. In this article, we have designed a hybrid system for RE. Benefiting from the complementary properties of feature-based methods, kernel-based methods and neural networks, the hybrid system could well combine lexical, syntactic, and semantic information, and therefore achieves significant improvements over the individual methods. To our knowledge, this is the first research that integrates the three methods into a uniform framework for RE.

The most immediate extension of our work is to improve the performance of CDR extraction by using additional biomedical knowledge bases. This can be done by constructing a knowledge-based system to include rich biomedical resources. Our future plan is to investigate the knowledge-based method to leverage more resources, and continue studying the hybrid approach to incorporate a wide variety of information.

## Funding

This research was supported by the National Natural Science Foundation of China (No. 61272375).
